# 

**Published:** 1902-02

**Authors:** 


### BOOKS RECEIVED.

DaCosta. Clinical Hematology. A Practical Guide to the Examination of the Blood with Reference to Diagnosis. By John C. DaCosta, Jr , M. D , Assistant Demonstrator of Clinical Medicine, Jefferson Medical College; Hematologist to the German Hospital, etc. Containing 8 full-page colored plates, 3 charts and 48 other illustrations. Octavo, 450 pages. Philadelphia: P. Blakiston's Son & Co., 1012 Walnut Street. 1901. [Price, $5.00 net.]

A Practical Treatise on Materia Medica and Therapeutics. With Especial Reference to the Clinical Application of Drugs. By John V. Shoemaker, M. D., LL D., Professor of Materia Medica, Pharmacology, Therapeutics and Clinical Medicine and Clinical Professor of Diseases of the Skin in the Medico-Chirurgical

College of Philadelphia; Physician to the Medico-Chirurgical Hospital; Member of the American Medical Association, of the Pennsylvania and Minnesota State Medical Societies, the American Academy of Medicine, the British Medical Association; Fellow of the Medical Society of London, etc., etc. Fifth edition, thoroughly revised. Pages viii.—1143 Size, 9¼ x 6¼ inches. Philadelphia: F. A. Davis Co., Publishers, 1914–16 Cherry Street. [Extra cloth, $5.00 net; sheep, $5.75 net, delivered.]

A Brief Manual of Prescription-Writing in Latin or English. For the use of Physicians, Pharmacists and Medical and Pharmacal Students. By M. L Neff, A M., M. D., Cedar Rapids, Ia. Pages v.—152. Size, 8 x 5¾ inches. Philadelphia: F. A. Davis Co., Publishers, 1914–16 Cherry Street. [Extra cloth, 75 cents net, delivered.]

Studies in the Psychology of Sex. Sexual Inversion. By Havelock Ellis, L.S.A., (England); Fellow of the Medico-Legal Society of New York and the Anthropological Society of Berlin; Honorary Fellow of the Chicago Academy of Medicine, etc.; General Editor of the Contemporary Science series since 1899. The "Studies in the Psychology of Sex" will probably be completed in five volumes. "Sexual Inversion" is second volume in the series. Pages xi.—272. Size, 8⅝ x 5¾ inches. Sold only to physicians, lawyers, advanced teachers and scientists. Philadelphia: F. A Davis Co., Publishers, 1914–16 Cherry Street. [Extra cloth, $2.00 net delivered.]

The Medical News Pocket Formulary. New (4th) edition. Containing 1,700 prescriptions, representing the latest and most approved methods of administering remedial agents. By E. Quin Thornton, M. D., Demonstrator of Therapeutics, Pharmacy and Materia Medica in the Jefferson Medical College, Philadelphia. Carefully revised to date of issue. In one wallet-shaped volume, strongly bound in leather, with pocket and pencil. Philadelphia and New York: Lea Brothers & Co. 1902. [Price, $1.50 net.]

Simon's Clinical Diagnosis. A Manual of Clinical Diagnosis by means of Microscopical and Chemical Methods. For Students, Hospital Physicians and Practitioners. By Charles E. Simon, M. D., author of Simon's Physiological Chemistry, etc. New (4th) edition, thoroughly revised and enlarged. In one handsome octavo volume of 608 pages, illustrated with 139 engravings and 19 plates in colors. Philadelphia and New York: Lea Brothers & Co. 1902. [Price, cloth, $3.75 net.]

Transactions of the American Gynecological Society. Vol. XXVI. For the year 1901 Published by the Society and edited by J. Riddle Goffe, M. D, Secretary. Philadelphia: William J. Dornan, Printer. 1901.

A Manual of Venereal and Sexual Diseases. By Wm. A. Hackett, Ph. G., M. C. P. S. (Ont.), Professor of Dermatology and Venereal Diseases, Michigan College of Medicine and Surgery; and N. E. Aronstane, M. D., Ph. G., Assistant in Chemistry and Clinical Dermatology in the same institution. Chicago: G. P. Engelhard & Co. 1901 [Price, $1.00.]

The Pocket Gray or Anatomist's Vade-Mecum. By the late Edward Cotterell, F. R. C. S. Fifth edition, revised and edited by C. H. Fogge, M. D, M. S. (Lond), F. K. C. S, Surgeon Demonstrator of Anatomy at Guy's Hospital. Twentieth thousand. New York: William Wood & Co. 1901. [Price, $ .]

The Practical Medical Series of Year Books. Ten volumes. Issued monthly, under the general editorial charge of Gustavus P. Head, M. D., Professor of Laryngology and Rhinology in the Chicago Post-Graduate Medical School. Vol. II. General Surgery. Edited by John B. Murphy, M. D., Professor of Surgery, Northwestern University Medical School. November, 1901. Pp. 515. Chicago: The Year Book Publishers, 40 Dearborn Street. [Cloth, $2.00.]

A System of Physiologic Therapeutics. A Practical Exposition of the Methods, other than Drug-giving, useful in the Prevention of Disease and in the Treatment of the Sick. Edited by Solomon Solis Cohen, A. M., M. D., Professor of Medicine and Therapeutics in the Philadelphia Polyclinic; Lecturer on Clinical Medicine at Jefferson Medical College; Physician to the Philadelphia Hospital and to the Rush Hospital for Consumption, etc. In eleven 8vo volumes. American, English, German and French authors. Volume VI. Dietotherapy and Food in Health. By Nathan S. Davis, Jr., A. M., M. D., Professor of the Principles and Practice of Medicine in Northwestern University Medical School; Physician to Mercy Hospital and Wesley Hospital, Chicago; Member American Medical Association, etc. Philadelphia: P. Blakiston's Son & Co , 1012 Walnut Street. 1901. [Price for the set complete, $27.50 net.]

Outlines of Anatomy. A Guide to the Methodical Study of the Human Body in the Dissecting Room. By Edmund W. Holms, A. B., M. D., Demonstrator of Anatomy, University of Pennsylvania (1892-1901); Surgeon to the Methodist-Episcopal Hospital, etc. Second edition. Lancaster, Pa.: The New Era Printing Co. 1902.

Manual of Physical Diagnosis. For the use of Students and Physicians. By James Tyson, M. D., Professor of Medicine in the University of Pennsylvania and Physician to the University Hospital; Physician to the Philadelphia Hospital; Fellow of the College of Physicians of Philadelphia; Member of the Association of American Physicians, etc. Fourth edition, revised and enlarged. Duodecimo. With colored and other illustrations. Philadelphia: P. Blakiston's Son & Co., 1012 Walnut Street. 1901. [Price, cloth, $1.50 net.]

American Edition of Nothnagel's Encyclopedia. Variola, Vaccination, Varicella, Cholera, Erysipelas, Whooping Cough, Hay Fever. Variola (including Vaccination). By Dr. H. Immermann, of Basle. Varicella. By Dr. Th. von Jürgensen, of Tübingen. Cholera Asiatic and Cholera Nostras. By Dr. C. Liebermeister, of Tübingen. Erysipelas and Erysipeloid. By Dr. H. Lenhartz, of Hamburg. Whooping Cough and Hay Fever. By Dr. G. Sticker, of Giessen. Edited, with additions, by Sir J. W. Moore, B. A., M. D., F. R. C. P. I., Professor of the Practice of Medicine, Royal College of Surgeons, Ireland. Handsome 8vo volume of 682 pages, illustrated. Philadelphia and London: W. B. Saunders & Co. 1902. [Price, cloth, $5.00 net; half-morocco, $6.00 net.]

